# Transmission Dynamics of Highly Pathogenic Avian Influenza A(H5N1) and A(H5N6) Viruses in Wild Birds, South Korea, 2023–2024

**DOI:** 10.3201/eid3108.250373

**Published:** 2025-08

**Authors:** Ye-Ram Seo, Andrew Y. Cho, Dong-Ju Kim, Young-Jae Si, Hye-sung Jeong, Su-woong Lee, Chang-Seon Song, Dong-Hun Lee

**Affiliations:** Konkuk University, Seoul, South Korea (Y.-R. Seo, A.Y. Cho, C.-S. Song, D.-H. Lee); National Institute of Wildlife Disease Control and Prevention, Gwangju, South Korea (D.-J. Kim, Y.-J. Si, H.-S. Jeong, S.-W Lee)

**Keywords:** Highly pathogenic avian influenza virus, influenza, viruses, respiratory infections, H5N1, H5N6, wild birds, South Korea, phylodynamics, phylogeography analysis, discrete trait analysis

## Abstract

We analyzed 15 cases of highly pathogenic avian influenza (HPAI) clade 2.3.4.4b virus infections detected in wild birds in South Korea during September 2023–March 2024. We isolated and sequenced 8 H5N1 and 7 H5N6 viruses. We investigated spatiotemporal transmission dynamics by using a Bayesian discrete trait phylodynamic model that incorporated geographic and host species information. Our source–sink dynamics support introductions of H5N1 viruses from northern Japan to South Korea and subsequent spread through multiple regions in South Korea. The H5N6 viruses were most likely introduced into southwestern South Korea and spread northeastward. Wild waterfowl, especially wild ducks, played a key role in transmission of both H5N1 and H5N6 viruses. Our data showed multiple introductions and extensive spread of HPAI clade 2.3.4.4b viruses and bidirectional transmission between Japan and South Korea. Our results highlight the value of enhanced active surveillance for monitoring HPAI viruses, which can provide insight into preventing future outbreaks.

Highly pathogenic avian influenza viruses (HPAIVs) cause severe clinical signs and high mortality rates in gallinaceous birds, leading to substantial economic losses in the poultry industry (*1*). Among HPAIVs, the A/Goose/Guangdong/1/1996 (gs/GD) lineage of H5Nx, which emerged in China in 1996, has caused outbreaks and diverged into 10 primary clades (nos. 0–9) and multiple subclades (*2*–*4*). Wild waterfowl play a crucial role in the wide and rapid geographic spread of gs/GD lineage highly pathogenic avian influenza (HPAI) H5Nx virus (*5*). Of note, HPAI H5Nx clade 2.3.4.4b viruses have caused widespread outbreaks across diverse geographic regions, including Asia, Europe, North America, South America, Africa, and even Antarctica (*6*–*10*). Increasing reports of HPAI clade 2.3.4.4b virus infections in diverse mammalian hosts, including dairy cows in North America, raise substantial public health concerns (*11*,*12*).

In South Korea, 6 major HPAI clade 2.3.4.4b outbreaks occurred during 2017–2024 (*13*–*16*). During the 2022–2023 HPAI outbreak, 174 cases of HPAI H5N1 clade 2.3.4.4b virus infection in various wild bird species were reported throughout South Korea (*17*). Spatiotemporal analysis of HPAI H5N1 clade 2.3.4.4b viruses revealed multiple hot spots in the Korean Peninsula that were responsible for the maintenance and spread of the viruses during the outbreak (*18*,*19*). Phylodynamic analysis integrating host trait information revealed a complex intertwined relationship between different regions inside and outside the Korean Peninsula and cross-species transmission of viruses among susceptible wild bird hosts (*17*,*20*). Whole-genome sequencing (WGS) of isolates from that outbreak also revealed emergence of diverse genotypes resulting from extensive reassortment (*21*).

During September 2023–March 2024, two different HPAI clade 2.3.4.4b virus subtypes, H5N1 and H5N6, caused influenza outbreaks in wild birds and poultry farms in South Korea (*22*–*24*). In particular, the index case in poultry was identified as a co-infection of H5N1 and H5N6 on a chicken farm (*24*). However, the evolutionary history and spread pattern of H5N1 and H5N6 viruses have not been clearly identified. To clarify the spatiotemporal diffusion and transmission dynamics between host species, we performed WGS on HPAIV isolates collected from wild birds during the 2023–2024 outbreak and performed a Bayesian phylodynamic analysis incorporating host species and sampling locations.

## Materials and Methods

### Virus Detection and Isolation

During September 2023–March 2024, the National Institute of Wildlife Disease Control and Prevention (NIWDC) of the Ministry of Environment of South Korea collected samples from wild birds as part of the national HPAI surveillance program. Samples were collected from wild bird feces (n = 11,294), carcasses (n = 555), and captured birds (n = 1,058) from 87 major migratory bird habitats across all provinces of South Korea. 

Oropharyngeal and cloacal swab samples from captured birds and carcasses and bird fecal samples were placed in phosphate-buffered saline with 0.1% volume of 400 mg/mL gentamicin and homogenized. We then filtered the supernatant by using a 0.45-µm Minisart Syringe Filter (Sartorius, https://www.sartorius.com) and inoculated into the allantoic cavity of 10-day-old specific pathogen–free embryonated chicken eggs. After 72 hours of incubation at 37°C, we harvested the allantoic fluids from eggs and tested for hemagglutination activity by using 10% chicken red blood cells. We extracted RNA from allantoic fluid samples positive for hemagglutination activity by using the Maxwell RSC Simply RNA Tissue Kit (Promega, https://www.promega.com) and screened for the avian influenza virus matrix (M) gene and H5 gene using real-time reverse transcription PCR (rRT-PCR) (*25*–*27*).

### WGS and Assembly

We sequenced 8 H5N1 and 7 H5N6 viruses in this study. We synthesized complementary DNA for M gene and H5 rRT-PCR–positive samples by using the SuperScript III First-Strand Synthesis System (Thermo Fisher Scientific, https://www.thermofisher.com). For samples confirmed as HPAIV via hemagglutinin (HA) gene sequencing, we amplified all 8 gene segments (HA, M, neuraminidase [NA], nucleoprotein [NP], nonstructural [NS], polymerase acidic [PA], and polymerase basic [PB] 1 and 2) by using AccuPrime Pfx DNA Polymerase (Invitrogen), according to methods described in a previous study (*28*). We constructed DNA libraries by using the Nextera DNA Flex Library Prep Kit (Illumina, https://www.illumina.com) and 96 dual-index barcodes, according to the manufacturer’s instruction. We conducted WGS on the MiSeq platform (Illumina) with 150 bp paired-end reads. We used CLC Genomics Workbench 24.0.1 software (QIAGEN, https://www.qiagen.com) to trim and assemble reads and identified HPAIV-positive samples (Table).

**Table:** Detailed information on highly pathogenic avian influenza A(H5N1) and A(H5N6) virus isolates from wild birds during 2023–2024 outbreak, in chronological order, South Korea*

No.†	Collection date	Sample ID	Region	Sample type	Host species	Subtype	Isolate ID
1	2023 Nov 27	23WS022-22	Jeollabuk-do	Captured	Eurasian wigeon	H5N1	EPI_ISL_18717640
2	2023 Dec 1	23WC066	Gyeongsangbuk-do	Carcass	Whooper swan	H5N1	EPI_ISL_20051148
3	2023 Dec 2	23WC068	Gyeongsangbuk-do	Carcass	Whooper swan	H5N1	EPI_ISL_20051147
4	2023 Dec 4	23WC069	Gyeongsangbuk-do	Carcass	Whooper swan	H5N1	EPI_ISL_20051146
7	2023 Dec 8	23WC075	Gyeongsangbuk-do	Carcass	Whooper swan	H5N6	EPI_ISL_18853568
9	2023 Dec 19	23WF435	Jeollabuk-do	Feces	Mandarin duck	H5N6	EPI_ISL_18853569
10	2023 Dec 21	23WC111	Gyeongsangbuk-do	Carcass	Bean goose	H5N6	EPI_ISL_18853650
11	2023 Dec 22	23WC116	Gyeongsangbuk-do	Carcass	Whooper swan	H5N6	EPI_ISL_18853651
12	2023 Dec 22	23WC117	Gyeongsangbuk-do	Carcass	Whooper swan	H5N1	EPI_ISL_20051145
13	2024 Jan 10	23WC160	Gyeongsangnam-do	Carcass	Bean goose	H5N6	EPI_ISL_20051144
14	2024 Jan 10	23WS033-1	Gwang-ju	Feces	Mandarin duck	H5N6	EPI_ISL_20051143
16	2024 Jan 26	23WC195	Jeju island	Carcass	Northern shoveler	H5N1	EPI_ISL_20051142
17	2024 Jan 30	23WC215	Jeju island	Carcass	Gadwall	H5N1	EPI_ISL_20051141
18	2024 Feb 4	23WC224	Gyeongsangbuk-do	Carcass	Peregrine falcon	H5N1	EPI_ISL_20051140
19	2024 Feb 6	23WC229	Gyeongsangnam-do	Carcass	Great cormorant	H5N6	EPI_ISL_20051139

*ID, identification. †Indicates the order of occurrence among the 19 confirmed cases of highly pathogenic avian influenza A(H5N1) clade 2.3.4.4b identified in wild birds during November 2023–February 2024, of which 15 viruses were detected and isolated by National Institute of Wildlife Disease Control and Prevention.

### Phylogenetic Analysis

To determine the genotypes and temporal signal of datasets for molecular clock analysis, we conducted maximum-likelihood analysis. We conducted BLAST searches (https://blast.ncbi.nlm.nih.gov) of all viral genomes sequenced in this study against the GISAID database (https://www.gisaid.org). We used the retrieved results as reference sequences for phylogenetic analysis. We used ElimDupes software (https://www.hiv.lanl.gov/content/sequence/elimdupesv2/elimdupes.html) to remove identical sequences. We aligned nucleotide sequences of each gene segment by using MAFFT version 7.490 (https://mafft.cbrc.jp). We constructed maximum-likelihood trees for each gene (PB2, PB1, PA, HA, NP, NA, M, and NS) by using RAxML version 8.0 (https://github.com/stamatak/standard-RAxML) and the general time-reversible model with 1,000 bootstrap iterations. We used iTOL (https://itol.embl.de) to visualize the trees and considered a cluster monophyletic only when it had a bootstrap support value >70 and a nucleotide sequence identity >97% (*29*).

We focused the phylodynamic analysis on the HA gene because of its variability and role as a key antigen. We extracted HA gene sequences belonging to same clade of our sequences from the maximum-likelihood phylogenetic tree. We used TempEst version 1.5.3 (http://tree.bio.ed.ac.uk/software/tempest) to conduct root-to-tip regression analysis and assess the temporal signal. Upon confirming a significant temporal signal (R^2^ >0.5), we used datasets to investigate transmission dynamics across geographic regions and host species. We conducted Bayesian discrete trait phylodynamic analyses of the HA gene by using BEAST version 1.10.4 (https://beast.community). We broadly categorized traits into host and region, and to reduce bias among traits, we performed subsampling, resulting in 6 major datasets (Appendix Table 1). For H5N1, we constructed 2 datasets for phylogeography. The discrete categories for estimation of international virus spread consisted of South Korea (n = 10), northern Japan (n = 10), central Japan (n = 6), southern Japan (n = 12), and outside of Korean Peninsula (i.e., Russia and China, n = 10). The discrete categories for estimation of virus spread between provinces within South Korea included Gyeong-buk (southeast province of South Korea, n = 5), Jeonbuk (southwest province of South Korea, n = 1), Jeonnam (south-southwest province of South Korea, n = 2), Jeju (southern island of South Korea, n = 3), and Japan (n = 10). Similarly, the regional dataset for H5N6 viruses included Gyeong-buk (n = 3), Gyeong-nam (south-southeast province of South Korea, n = 2), Jeonbuk (n = 1), Jeonnam (n = 3), and Japan (n = 1).

For datasets analyzing transmission among hosts, we categorized H5N1 sequences into raptors (n = 1), domestic ducks (n = 2), and wild waterfowl (n = 8) from South Korea and wild waterfowl (n = 4) and crows (n = 6) from Japan. The H5N6 sequence dataset included domestic ducks (n = 2) and wild waterfowl (n = 7) from South Korea, 1 raptor from Japan, and H5N1 sequences from East Asia collected during 2022–2023 (n = 36). We categorized the viruses identified from East Asia during 2022–2023 as a discrete nominal category regardless of animal species and sampling location because the viruses from wild birds and poultry across that region during the 2022–2023 epidemic were the most probable ancestral origins inferred from the ML phylogenetic analysis. To elucidate the role of wild waterfowl in transmission, we combined H5N1 and H5N6 data to form datasets comprising wild ducks (n = 8), geese (n = 9), swans (n = 8), and other hosts (n = 10).

For Bayesian inferences, we applied a Hasegawa-Kishino-Yano substitution model plus gamma, an uncorrelated log-normal distribution, and a Gaussian Markov random field Bayesian skyride coalescent prior (*30*). We executed Markov chain Monte Carlo runs of the configuration in parallel across 3 separate chains, each consisting of 100 million steps. We combined samples from those chains after a 10% burn-in period. We used Tracer version 1.5 (https://beast.community/tracer) to analyze parameters with adequate effective sample sizes (>200). We generated a maximum clade credibility tree by using TreeAnnotator (https://beast.community/treeannotator) and visualized the tree by using FigTree version 1.4.4 (http://tree.bio.ed.ac.uk/software/Figtree). To quantify the support for transmission routes, we used SpreaD3 version 1.0.7 (https://beast.community/spread3) and interpreted results as positive support when the Bayes factor (BF) was >3 and the posterior probability (PP) was >0.5 and strong support when the BF was >20, and the PP was >0.9 (*31*). We also used FluMutGUI 3.1.1 (https://github.com/izsvenezie-virology/FluMutGUI) to identify molecular markers for mammalian adaptation across the 8 viral genes.

## Results

### Overview of 2023–2024 HPAI viruses from Wild Birds in South Korea

**Figure 1:**
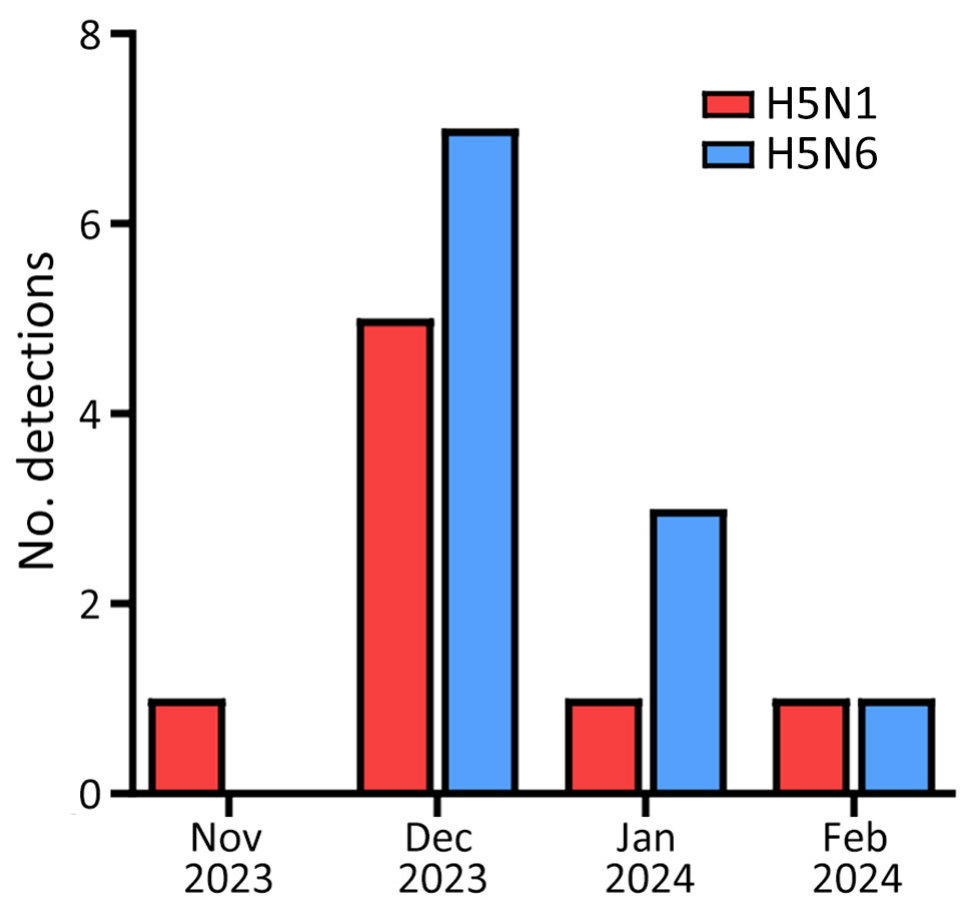
Number of detections per month in a study of transmission dynamics of highly pathogenic avian influenza A(H5N1) and A(H5N6) viruses in wild birds, South Korea, 2023–2024.

During November 27, 2023–February 6, 2024, a total of 8 cases of H5N1 and 11 cases of H5N6 were reported from wild birds in South Korea (*22*,*23*) (Appendix Figure 1). Among those cases, we isolated 8 H5N1 and 7 H5N6 viruses (Table). Next-generation sequencing yielded total read counts ranging from 21,507 to 756,810 and average coverage depths ranging from 240.80 to 8,442.09. During the 2023–2024 winter season, HPAI H5N1 was detected in a Eurasian wigeon (*Mareca penelope*) on November 27, 2023, six days before the initial H5N1 and H5N6 outbreak in poultry. The index H5N6 was detected in a Mandarin duck (*Aix galericulata*) on December 4, 2023. The number of cases gradually increased over time, reaching a peak in December 2023 (Figure 1).

### Origin and Genotypes of H5N1 and H5N6 HPAI Viruses

Maximum-likelihood phylogenetic analysis of the 8 genes revealed that the HA and M genes of H5N1 and H5N6 shared >97% nucleotide sequence identity and formed a monophyletic cluster. The other 5 internal genes of isolates from South Korea formed distinct monophyletic clades within their respective subtypes (Appendix Figures 2–9). The phylogenies showed that the H5N1 viruses consisted of genes derived from HPAI H5N1 strains previously circulating in East Asia. In contrast, the H5N6 viruses were reassortants between PB2, PA, NP, and NS genes from low pathogenicity avian influenza viruses from Eurasia and the NA gene from H5N6 viruses identified in China. Those findings were consistent with findings observed in genetic analysis of index cases (*22*,*23*), suggesting no further reassortment occurred in wild birds during that outbreak.

According to recommendations from the European Food Safety Authority (*32*), we screened 14 selections of molecular markers associated with the pandemic potential of avian influenza viruses (HA, 222L; PB2, 271A, 292V, 526R, 588V, 591K, 627K, 627V, 631L, and 701N; PA, 356R; NP, 52N; and MP, 95K) by using the deduced amino acid sequences of all 15 isolates. We analyzed mammalian adaptation markers, but did not detect major markers (PB2: E627K, D701N), and we identified only a few minor markers (Appendix Table 2). Among other minor mutations, we observed 156A in HA, which is associated with increased binding to α2,6-sialic acid, and N66S in PB1-F2, which is associated with increased virulence and replication in mice.

### Transmission Dynamics of HPAI H5N1 Viruses in South Korea during 2023–2024

**Figure 2:**
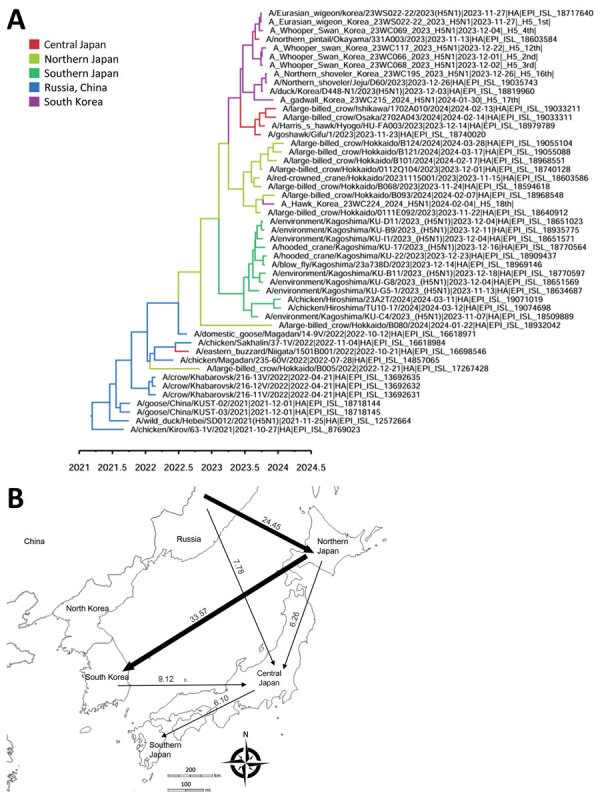
Discrete phylogeographic reconstruction of diffusion dynamics of highly pathogenic avian influenza A(H5N1) virus in wild birds, East Asia, 2023–2024. A) Maximum clade credibility tree constructed by using the hemagglutinin gene of H5N1 viruses. Each branch is colored according to the geographic location. GISAID (https://www.gisaid.org) accession numbers are shown. Scale bar shows years of detection in decimal year format. B) Visualization of transmission dynamics inferred by using the geographic location trait of countries adjacent to South Korea. Arrows represent the direction of the viral transmission; annotated values along arrows represent Bayes factors. Thick arrows indicate strongly supported routes (Bayes factor >20, posterior probability >0.8). Maps provided by d-maps.com (https://d-maps.com).

**Figure 3:**
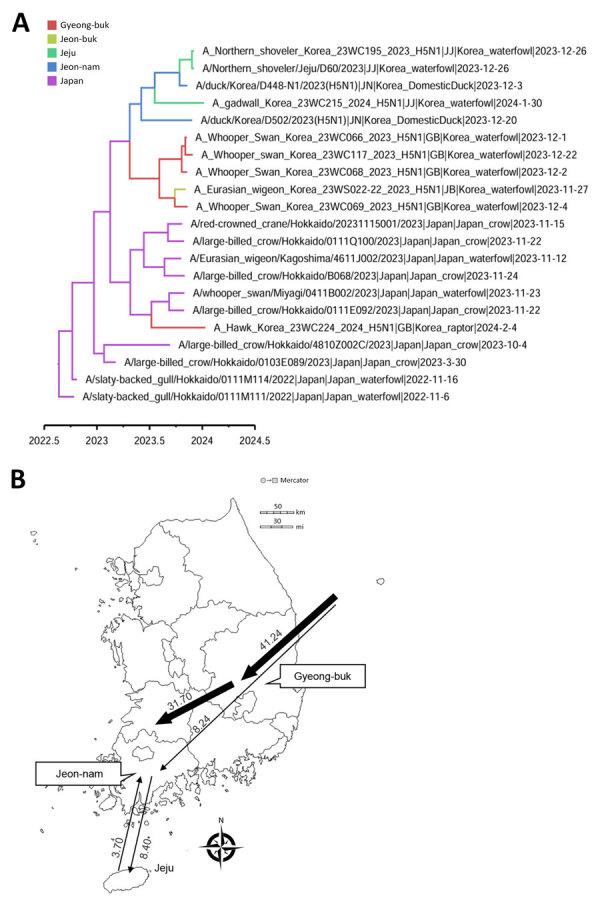
Transmission dynamics of highly pathogenic avian influenza A(H5N1) virus in wild birds, South Korea, 2023–2024. A) Maximum clade credibility tree constructed using the hemagglutinin gene of H5N1 viruses. Each branch is colored according to the geographic location. Scale bar shows years of detection in decimal year format. B) Visualization of transmission dynamics inferred by using the geographic location trait in South Korea. Arrows represent the direction of the viral transmission; annotated values along arrows represent Bayes factors. Thick arrows indicate strongly supported routes (Bayes factor >20, posterior probability >0.8). Maps provided by d-maps.com (https://d-maps.com).

**Figure 4:**
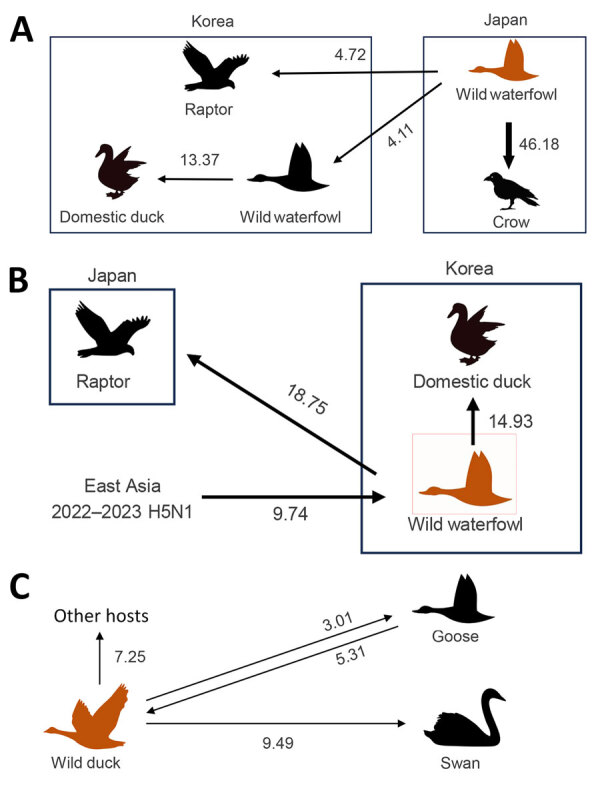
Transmission dynamics of highly pathogenic avian influenza (HPAI) A(H5N1) and A(H5N6) viruses in wild birds, South Korea and Asia, 2023–2024. A, B) Transmission dynamics inferred using the hemagglutinin gene of H5N1 (A) and H5N6 (B) viruses, incorporating the host trait. C) Transmission dynamics inferred using the hemagglutinin genes of both HPAI H5N1) clade 2.3.4.4b and H5N6 viruses. Arrows represent the direction of the viral transmission; annotated values represent Bayes factors. Thick arrow indicates a strongly supported route (Bayes factor >20, posterior probability >0.8). Orange indicates the largest source trait.

The maximum clade credibility phylogeny constructed from the HA gene of HPAI H5N1 viruses suggested that the virus initially entered northern Japan from China or Russia, then subsequently spread to central Japan and South Korea. Within Japan, the virus spread southward from the northern region to the southern region (Figure 2). In South Korea, we identified at least 2 separate H5N1 virus introductions, which most likely entered through the east-central region (Gyeong-buk province) and the southwest region (Jeon-nam province). The virus subsequently spread southwestward (Jeon-buk) and, finally, to Jeju Island in southern South Korea (Figure 3). Of note, within South Korea, virus dissemination from northern Japan to South Korea (BF 33.57, PP 0.91), from Japan to Gyeong-buk (BF 41.24, PP 0.926), and from Gyeong-buk to Jeon-buk (BF 31.701, PP 0.906) were among the most probable H5N1 transmission routes (BF >30 and high support values) (Appendix Tables 3, 4). Our findings suggest the virus was transmitted from Japan to South Korea through migratory wild waterfowl (Figure 4, panels A, B; Appendix Table 5). In particular, H5N1 virus was transmitted from wild waterfowl to raptors (BF 4.725, PP 0.591) and domestic ducks (BF 13.376, PP 0.803) in South Korea, as well as to crows in Japan (BF 46.186, PP 0.934) (Figure 4, panel A). We also estimated source–sink dynamics between wild waterfowl, including wild ducks, geese, swans, and other wild waterfowl. Our data suggest that wild ducks played a major role in transmitting the virus to other hosts (Figure 4, panel C).

### Transmission Dynamics of HPAI H5N6 Viruses in South Korea during 2023–2024

**Figure 5:**
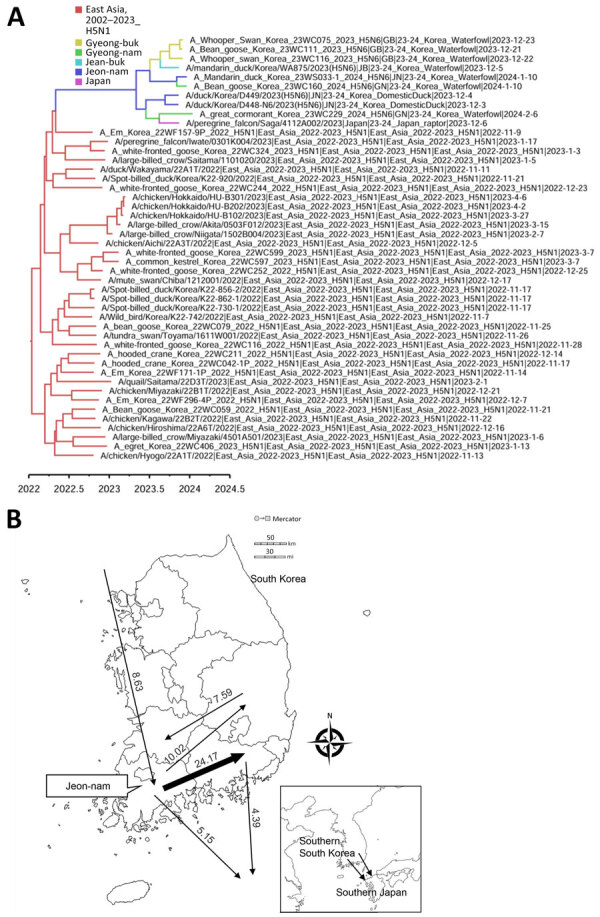
Discrete phylogeographic reconstruction of diffusion dynamics of influenza A(H5N6) viruses in East Asia during 2022–2023 used in a study of transmission dynamics of highly pathogenic avian influenza A(H5N1) and A(H5N6) viruses in wild birds, South Korea, 2023–2024. A) Maximum clade credibility tree constructed using the hemagglutinin gene of H5N6 viruses. Each branch is colored according to the geographic location. Scale bar shows years of detection in decimal year format. B) Visualization of transmission dynamics inferred by using the geographic location traits within South Korea. Arrows represent the direction of the viral transmission; annotated values represent Bayes factors. Thick arrow indicates a strongly supported route (Bayes factor >20, posterior probability >0.8). Maps provided by d-maps.com (https://d-maps.com).

The HA gene of HPAI H5N6 isolated during 2023–2024 was highly similar to that of the HPAI H5N1 viruses circulating in northeast Asia during the 2022–23 winter season (*23*). Phylogenetic analysis suggested that, after reassortment with the N6 gene originating in China, H5N6 likely entered the southwestern region of the Korean Peninsula (Jeonnam) and subsequently spread northeastward (Gyeong-buk and Gyeong-nam). Our findings also supported transmission from southern South Korea (Jeon-nam and Gyeong-nam) to southern Japan (Figure 5). Among the various HPAI H5N6 transmission routes, our findings supported movement from Jeonnam to Gyeong-nam (BF 24.176, PP 0.850) and Gyeong-buk (BF 10.022, PP 0.701) (Appendix Table 6). For virus transmission between host species, H5N6 most likely was transmitted from South Korea to Japan via wild waterfowl (Figure 4, panel C). Our findings supported virus spread from wild waterfowl to raptors in Japan (BF 18.752, PP 0.893) and to domestic ducks in South Korea (BF 14.932, PP 0.869). Consistent with the H5N1 viruses, wild ducks played the most prominent role in transmission to other species (Figure 4; Appendix Table 7).

## Discussion

Over the past decade, molecular epidemiologic studies in South Korea have helped clarify the genetic diversity and transmission dynamics of HPAI clade 2.3.4.4 viruses (*16*,*33*). Genomic sequencing and phylodynamic analysis have shown that, since 2014, multiple introductions of reassortant HPAI H5Nx clade 2.3.4.4 viruses by wild waterfowl have occurred almost every fall migration season in South Korea, and then viral detections gradually decrease or disappear within ≈5 months, around the end of waterfowl migration season (*13*–*15*,*34*,*35*). Previous phylogeography studies on HPAIV outbreaks in South Korea and Japan during 2022–2023 and 2023–2024 revealed bidirectional virus exchange between those countries (*17*,*22*,*23*). Consistent with those findings, our data also highlight the bidirectional virus exchange between South Korea and Japan. In November 2023, HPAI H5N1 viruses initially entered South Korea’s Gyeongbuk and Jeonnam regions from northern Japan and subsequently spread southwestward. Given that H5N1 virus was dominant early in the season, that spread likely was associated with the southward movement of migratory birds in both South Korea and Japan during the early phase of the season. In addition, movement of H5N1 from the mid-latitude regions of South Korea to central Japan follows a pattern observed in previous seasons, suggesting that transmission might have occurred between regions at similar latitudes (*17*).

In December 2023, HPAI H5N6 appears to have entered the Jeonnam region and displayed a more irregular transmission pattern than H5N1, likely influenced by movement of wild birds within their wintering sites. Furthermore, given its introduction into the Jeonnam region, H5N6, unlike H5N1, likely was not introduced from Japan but rather from proximal countries to the west, such as China or Russia. We also observed a notable transmission link between southern Japan and southern South Korea, resembling patterns of viral movements from previous seasons where transmission occurred through hooded cranes (*Grus monacha*) in southern Japan and southern South Korea (*17*,*36*).

Migratory waterfowl disseminate HPAIVs during fall migration through north-to-south migration routes (*33*,*37*–*39*), including wild ducks (*40*), geese (*41*), and swans (*37*) that migrate from Siberia to South Korea and Japan. Those species share stopover and wintering habitats around inland water bodies and play a crucial role in the maintenance and transmission of HPAIVs. In this study, we largely attributed the diffusion of H5N1 and H5N6 viruses to wild waterfowl. Our findings indicate that wild ducks played a major role in virus transmission not only to other wild waterfowl species, including geese and swans, but also to crows, raptors, and domestic ducks. During the outbreak, whooper swans (*Cygnus cygnus*) accounted for the highest (44.45%) percentage of H5N1 cases among wild birds in South Korea, which might be because of their high susceptibility to HPAIVs and distinctive morphology (*37*). During outbreaks in South Korea, we also detected HPAIVs from raptors that likely were infected by hunting infected birds or scavenging virus-contaminated carcasses (*17*,*42*). Of note, we detected H5N6 virus from a great cormorant (*Phalacrocorax carbo*) found dead. The great cormorant used to breed in Primorsky Krai and Sakhalin, Russia, and descend to South Korea and Japan every winter but is now an invasive species in South Korea, where it has been endemic since the 2000s because of the effects of climate change; the current population is estimated to be 23,000–30,000 (*43*). HPAI virus infection in this new waterfowl population is a concern because it can substantially affect the epidemiology and ecology of the virus.

Since 2014, HPAI clade 2.3.4.4 viruses have evolved through reassortment with prevailing local low pathogenicity avian influenza viruses (*44*). A wide range of avian species, including wild and domestic waterfowl, appear to be permissive for infection and transmission of clade 2.3.4.4 viruses. Among those species, domestic ducks play a key role in the maintenance, amplification, and spread of HPAIVs of wild bird origin to terrestrial poultry (*45*). In this study, estimation of the host transmission dynamics supports that H5N1 and H5N6 viruses are transmitted from wild waterfowl to domestic ducks in South Korea. Because domestic ducks can host a variety of avian influenza viruses as a natural reservoir species, that population can accelerate the genetic and antigenic evolution of viruses, potentially giving rise to new strains with altered antigenicity, pathogenicity, or increased zoonotic potential. To prevent dissemination of HPAI from wild birds to poultry, biosecurity measures should be enhanced at poultry farms, especially those located near wild bird habitats, to block contact with wild birds or their excreta.

To minimize the impact of HPAIV in wild and domestic animals, effective information sharing among countries along migratory bird flyways and timely reporting of genomic surveillance data are essential. Next-generation sequencing–based genomic surveillance activities enable rapid and accurate characterization of complete viral genome and evolutionary history of viruses (*46*–*48*). Despite those advances and the high number of HPAIV cases reported in Eurasia in recent years, the amount of complete genome sequence data available in public databases was limited in terms of representativeness across different countries and species. In particular, the limited availability of recent genomic sequence data from poultry outbreaks could hinder the accurate reconstruction of transmission dynamics at the wildlife–domestic poultry interface in South Korea. The limited sample sizes for certain discrete traits in this study might have introduced unrecognized biases in the inferred transmission dynamics. Nonetheless, our findings underscore the need for enhanced genomic sequencing and rapid sharing of poultry-derived viral sequences to better track viral evolution and spread. 

In conclusion, public sharing of genome sequence data varies substantially between different countries and laboratories (*49*,*50*). In the last few years, we have tried to rapidly provide updated information on HPAIVs identified in wild birds in South Korea by generating and sharing HPAIV sequence data from extensive genomic surveillance efforts conducted by NIWDC (*16*,*17*,*22*,*23*). Enhanced genomic surveillance in both wild and domestic animals are needed to monitor evolution and spread of HPAIVs, which can provide insights into preventing future outbreaks and assessing zoonotic potential.

Appendix Additional information on transmission dynamics of highly pathogenic avian influenza A(H5N1) and A(H5N6) viruses in wild birds, South Korea, 2023–2024.
