# Supplementary material for: Transmission Dynamics of Highly Pathogenic Avian Influenza A(H5N1) and A(H5N6) Viruses in Wild Birds, South Korea, 2023–2024
Source: Emerg Infect Dis. 2025 Aug;31(8):1561–72. doi: 10.3201/eid3108.250373 (PMC12309777; doi:10.3201/eid3108.250373)
Supplement: Appendix — Additional information on transmission dynamics of highly pathogenic avian influenza A(H5N1) and A(H5N6) viruses in wild birds, South Korea, 2023–2024. [file 25-0373-Techapp-s1.pdf]

Article DOI: https://doi.org/10.3201/eid3108.250373

EID cannot ensure accessibility for supplementary materials supplied by authors. Readers who have difficulty accessing supplementary content should contact the authors for assistance.

Transmission Dynamics of Highly Pathogenic Avian Influenza A(H5N1) and A(H5N6) Viruses in Wild Birds, South Korea, 2023–2024

Appendix

Appendix Table 1. List of sequences and traits by dataset type used in discrete trait analysis.

Dataset	Trait	Strain	GISAID isolate ID
H5N1 Location 1 (n = 21)	Gyeong-buk	A_Whooper_Swan_Korea_23WC066_2023_H5N1	EPI_ISL_20051148
		A_Whooper_swan_Korea_23WC117_2023_H5N1	EPI_ISL_20051145
		A_Whooper_Swan_Korea_23WC068_2023_H5N1	EPI_ISL_20051147
		A_Whooper_Swan_Korea_23WC069_2023_H5N1	EPI_ISL_20051146
		A_Peregrine Falcon_Korea_23WC224_2024_H5N1	EPI_ISL_20051140
	Jeju	A_Northern_shoveler_Korea_23WC195_2023_H5N1	EPI_ISL_20051142
		A/Northern_shoveler/Jeju/D60/2023	EPI_ISL_19035743
		A_gadwall_Korea_23WC215_2024_H5N1	EPI_ISL_20051141
	Jeon-buk	A_Eurasian_wigeon_Korea_23WS022–22_2023_H5N1	EPI_ISL_18717640
	Jeon-nam	A/duck/Korea/D448-N1/2023(H5N1)	EPI_ISL_18819960
		A/duck/Korea/D502/2023(H5N1)	EPI_ISL_18819797
	Japan	A/red-crowned_crane/Hokkaido/20231115001/2023	EPI_ISL_18603586
		A/large-billed_crow/Hokkaido/0111Q100/2023	EPI_ISL_18640907
		A/Eurasian_wigeon/Kagoshima/4611J002/2023	EPI_ISL_18603583
		A/large-billed_crow/Hokkaido/B068/2023	EPI_ISL_18594618
		A/whooper_swan/Miyagi/0411B002/2023	EPI_ISL_18640911
		A/large-billed_crow/Hokkaido/0111E092/2023	EPI_ISL_18640912
		A/large-billed_crow/Hokkaido/4810Z002C/2023	EPI_ISL_18505897
		A/large-billed_crow/Hokkaido/0103E089/2023	EPI_ISL_17950253
		A/slaty-backed_gull/Hokkaido/0111M114/2022	EPI_ISL_16955798
		A/slaty-backed_gull/Hokkaido/0111M111/2022	EPI_ISL_16698579
H5N1 Location 2 (n = 48)	Southern Japan	A/hooded_crane/Kagoshima/KU-17/2023_(H5N1)	EPI_ISL_18770564
		A/environment/Kagoshima/KU-B9/2023_(H5N1)	EPI_ISL_18935775
		A/hooded_crane/Kagoshima/KU-22/2023	EPI_ISL_18909437
		A/environment/Kagoshima/KU-C4/2023_(H5N1)	EPI_ISL_18509889
		A/environment/Kagoshima/KU-G8/2023_(H5N1)	EPI_ISL_18651569
		A/chicken/Hiroshima/23A2T/2024	EPI_ISL_19071019
		A/environment/Kagoshima/KU-D11/2023_(H5N1)	EPI_ISL_18651023
		A/blow_fly/Kagoshima/23a738D/2023	EPI_ISL_18969146
		A/chicken/Hiroshima/TU10–17/2024	EPI_ISL_19074698
		A/environment/Kagoshima/KU-I1/2023_(H5N1)	EPI_ISL_18651571
		A/environment/Kagoshima/KU-B11/2023_(H5N1)	EPI_ISL_18770597
		A/environment/Kagoshima/KU-G5–1/2023_(H5N1)	EPI_ISL_18634687
	Central Japan	A/large-billed_crow/Ishikawa/1702A010/2024	EPI_ISL_19033211
		A/northern_pintail/Okayama/331A003/2023	EPI_ISL_18603584
		A/Harris_s_hawk/Hyogo/HU-FA003/2023	EPI_ISL_18979789
		A/eastern_buzzard/Niigata/1501B001/2022	EPI_ISL_16698546
		A/goshawk/Gifu/1/2023	EPI_ISL_18740020
		A/large-billed_crow/Osaka/2702A043/2024	EPI_ISL_19033311
	Northern Japan	A/large-billed_crow/Hokkaido/0111E092/2023	EPI_ISL_18640912

Dataset	Trait	Strain	GISAID isolate ID
		A/large-billed_crow/Hokkaido/B080/2024	EPI_ISL_18932042
		A/large-billed_crow/Hokkaido/B124/2024	EPI_ISL_19055104
		A/large-billed_crow/Hokkaido/B068/2023	EPI_ISL_18594618
		A/large-billed_crow/Hokkaido/0112Q104/2023	EPI_ISL_18740128
		A/large-billed_crow/Hokkaido/B005/2022	EPI_ISL_17267428
		A/large-billed_crow/Hokkaido/B093/2024	EPI_ISL_18968548
		A/large-billed_crow/Hokkaido/B121/2024	EPI_ISL_19055088
		A/large-billed_crow/Hokkaido/B101/2024	EPI_ISL_18968551
		A/red-crowned_crane/Hokkaido/20231115001/2023	EPI_ISL_18603586
	Outside of Korea	A/chicken/Magadan/235–60V/2022	EPI_ISL_14857065
		A/chicken/Kirov/63–1V/2021	EPI_ISL_8769023
		A/crow/Khabarovsk/216–11V/2022	EPI_ISL_13692630
		A/goose/People’s Republic of China/KUST-03/2021	EPI_ISL_18718145
		A/crow/Khabarovsk/216–13V/2022	EPI_ISL_13692634
		A/goose/People’s Republic of China/KUST-02/2021	EPI_ISL_18718144
		A/domestic_goose/Magadan/14–9V/2022	EPI_ISL_16618970
		A/crow/Khabarovsk/216–12V/2022	EPI_ISL_13692632
		A/chicken/Sakhalin/37–1V/2022	EPI_ISL_16618984
		A/wild_duck/Hebei/SD012/2021(H5N1)	EPI_ISL_12572664
	South Korea	A_Whooper_Swan_Korea_23WC069_2023_H5N1	EPI_ISL_20051146
		A_Whooper_Swan_Korea_23WC068_2023_H5N1	EPI_ISL_20051147
		A/duck/Korea/D448-N1/2023(H5N1)	EPI_ISL_18819960
		A_Peregrine Falcon_Korea_23WC224_2024_H5N1	EPI_ISL_20051140
		A_Eurasian_wigeon_Korea_23WS022–22_2023_H5N1	EPI_ISL_18717640
		A_Northern_shoveler_Korea_23WC195_2023_H5N1	EPI_ISL_20051142
		A/Northern_shoveler/Jeju/D60/2023	EPI_ISL_19035743
		A_gadwall_Korea_23WC215_2024_H5N1	EPI_ISL_20051141
		A_Whooper_Swan_Korea_23WC066_2023_H5N1	EPI_ISL_20051148
		A_Whooper_swan_Korea_23WC117_2023_H5N1	EPI_ISL_20051145
H5N6 Location (n = 46)	East Asia 2022–2023 H5N1	A_Em_Korea_22WF157–9P_2022_H5N1	EPI_ISL_18245277
		A/peregrine_falcon/Iwate/0301K004/2023	EPI_ISL_17309160
		A_white-fronted_goose_Korea_22WC324_2023_H5N1	EPI_ISL_18245344
		A/large-billed_crow/Saitama/1101020/2023	EPI_ISL_18130247
		A/duck/Wakayama/22A1T/2022	EPI_ISL_18284467
		A/Spot-billed_duck/Korea/K22–920/2022	EPI_ISL_15944667
		A_white-fronted_goose_Korea_22WC244_2022_H5N1	EPI_ISL_18245328
		A/chicken/Hokkaido/HU-B301/2023	EPI_ISL_17638448
		A/chicken/Hokkaido/HU-B202/2023	EPI_ISL_17638143
		A/chicken/Hokkaido/HU-B102/2023	EPI_ISL_17638141
		A/large-billed_crow/Akita/0503F012/2023	EPI_ISL_18007231
		A/large-billed_crow/Niigata/1502B004/2023	EPI_ISL_17949986
		A/chicken/Aichi/22A3T/2022	EPI_ISL_18284468
		A_white-fronted_goose_Korea_22WC599_2023_H5N1	EPI_ISL_18245383
		A_common_kestrel_Korea_22WC597_2023_H5N1	EPI_ISL_18245382
		A_white-fronted_goose_Korea_22WC252_2022_H5N1	EPI_ISL_18245332
		A/mute_swan/Chiba/1212001/2022	EPI_ISL_16955767
		A/Spot-billed_duck/Korea/K22–856–2/2022	EPI_ISL_15944663
		A/Spot-billed_duck/Korea/K22–862–1/2022	EPI_ISL_15944665
		A/Spot-billed_duck/Korea/K22–730–1/2022	EPI_ISL_15943002
		A/Wild_bird/Korea/K22–742/2022	EPI_ISL_15943015
		A_bean_goose_Korea_22WC079_2022_H5N1	EPI_ISL_18245303
		A/tundra_swan/Toyama/1611W001/2022	EPI_ISL_17949987
		A_white-fronted_goose_Korea_22WC116_2022_H5N1	EPI_ISL_18245307
		A_hooded_crane_Korea_22WC211_2022_H5N1	EPI_ISL_18245322
		A_hooded_crane_Korea_22WC042–1P_2022_H5N1	EPI_ISL_18245290
		A_Em_Korea_22WF171–1P_2022_H5N1	EPI_ISL_18245284
		A/quail/Saitama/22D3T/2023	EPI_ISL_18284478
		A/chicken/Miyazaki/22B1T/2022	EPI_ISL_18284475
		A_Em_Korea_22WF296–4P_2022_H5N1	EPI_ISL_18245317
		A_Bean_goose_Korea_22WC059_2022_H5N1	EPI_ISL_18245288
		A/chicken/Kagawa/22B2T/2022	EPI_ISL_18284469
		A/chicken/Hiroshima/22A6T/2022	EPI_ISL_18284473
		A/large-billed_crow/Miyazaki/4501A501/2023	EPI_ISL_18066450
		A_egret_Korea_22WC406_2023_H5N1	EPI_ISL_18245360
		A/chicken/Hyogo/22A1T/2022	EPI_ISL_18284463
	Japan	A/peregrine_falcon/Saga/4112A002/2023	EPI_ISL_18740267
	Gyeong-buk	A_Whooper_Swan_Korea_23WC075_2023_H5N6	EPI_ISL_18853568
		A_Bean_goose_Korea_23WC111_2023_H5N6	EPI_ISL_18853650
		A_Whooper_swan_Korea_23WC116_2023_H5N6	EPI_ISL_18853651

Dataset	Trait	Strain	GISAID isolate ID
	Gyeong-nam	A_Bean_goose_Korea_23WC160_2024_H5N6	EPI_ISL_20051144
		A_great_cormorant_Korea_23WC229_2024_H5N6	EPI_ISL_20051139
	Jeon-buk	A/mandarin_duck/Korea/WA875/2023(H5N6)	EPI_ISL_18819826
	Jeon-nam	A_Mandarin_duck_Korea_23WS033–1_2024_H5N6	EPI_ISL_20051143
		A/duck/Korea/D449/2023(H5N6)	EPI_ISL_18819961
		A/duck/Korea/D448-N6/2023(H5N6)	EPI_ISL_18819959
H5N1 host (n = 21)	Japanese crow	A/red-crowned_crane/Hokkaido/20231115001/2023	EPI_ISL_18603586
		A/large-billed_crow/Hokkaido/0111Q100/2023	EPI_ISL_18640907
		A/large-billed_crow/Hokkaido/B068/2023	EPI_ISL_18594618
		A/large-billed_crow/Hokkaido/0111E092/2023	EPI_ISL_18640912
		A/large-billed_crow/Hokkaido/4810Z002C/2023	EPI_ISL_18505897
		A/large-billed_crow/Hokkaido/0103E089/2023	EPI_ISL_17950253
	Japanese wild waterfowl	A/Eurasian_wigeon/Kagoshima/4611J002/2023	EPI_ISL_18603583
		A/whooper_swan/Miyagi/0411B002/2023	EPI_ISL_18640911
		A/slaty-backed_gull/Hokkaido/0111M114/2022	EPI_ISL_16955798
		A/slaty-backed_gull/Hokkaido/0111M111/2022	EPI_ISL_16698579
	Korean domestic duck	A/duck/Korea/D502/2023(H5N1)	EPI_ISL_18819797
		A/duck/Korea/D448-N1/2023(H5N1)	EPI_ISL_18819960
	Korean raptor	A_Peregrine Falcon_Korea_23WC224_2024_H5N1	EPI_ISL_20051140
	Korean wild waterfowl	A_Whooper_Swan_Korea_23WC066_2023_H5N1	EPI_ISL_20051148
		A_Whooper_swan_Korea_23WC117_2023_H5N1	EPI_ISL_20051145
		A_Whooper_Swan_Korea_23WC068_2023_H5N1	EPI_ISL_20051147
		A_Whooper_Swan_Korea_23WC069_2023_H5N1	EPI_ISL_20051146
		A_Eurasian_wigeon_Korea_23WS022–22_2023_H5N1	EPI_ISL_18717640
		A/Northern_shoveler/Jeju/D60/2023	EPI_ISL_19035743
		A_Northern_shoveler_Korea_23WC195_2023_H5N1	EPI_ISL_20051142
		A_gadwall_Korea_23WC215_2024_H5N1	EPI_ISL_20051141
H5N6 host (n = 46)	23–24 Japanese raptor	A/peregrine_falcon/Saga/4112A002/2023	EPI_ISL_18740267
	23–24 Korean domestic duck	A/duck/Korea/D449/2023(H5N6)	EPI_ISL_18819961
		A/duck/Korea/D448-N6/2023(H5N6)	EPI_ISL_18819959
	23–24 Korean wild waterfowl	A_Whooper_swan_Korea_23WC116_2023_H5N6	EPI_ISL_18853651
		A/mandarin_duck/Korea/WA875/2023(H5N6)	EPI_ISL_18819826
		A_Whooper_Swan_Korea_23WC075_2023_H5N6	EPI_ISL_18853568
		A_Bean_goose_Korea_23WC111_2023_H5N6	EPI_ISL_18853650
		A_Mandarin_duck_Korea_23WS033–1_2024_H5N6	EPI_ISL_20051143
		A_Bean_goose_Korea_23WC160_2024_H5N6	EPI_ISL_20051144
		A_great_cormorant_Korea_23WC229_2024_H5N6	EPI_ISL_20051139
	East Asia 2022–2023 H5N1	A_Em_Korea_22WF296–4P_2022_H5N1	EPI_ISL_18245317
		A/chicken/Miyazaki/22B1T/2022	EPI_ISL_18284475
		A/chicken/Hyogo/22A1T/2022	EPI_ISL_18284463
		A_egret_Korea_22WC406_2023_H5N1	EPI_ISL_18245360
		A/large-billed_crow/Miyazaki/4501A501/2023	EPI_ISL_18066450
		A/chicken/Kagawa/22B2T/2022	EPI_ISL_18284469
		A_Bean_goose_Korea_22WC059_2022_H5N1	EPI_ISL_18245288
		A/chicken/Hiroshima/22A6T/2022	EPI_ISL_18284473
		A_hooded_crane_Korea_22WC042–1P_2022_H5N1	EPI_ISL_18245290
		A_hooded_crane_Korea_22WC211_2022_H5N1	EPI_ISL_18245322
		A/quail/Saitama/22D3T/2023	EPI_ISL_18284478
		A_Em_Korea_22WF171–1P_2022_H5N1	EPI_ISL_18245294
		A/chicken/Hokkaido/HU-B202/2023	EPI_ISL_17638143
		A/chicken/Hokkaido/HU-B301/2023	EPI_ISL_17638448
		A/chicken/Hokkaido/HU-B102/2023	EPI_ISL_17638141
		A/large-billed_crow/Akita/0503F012/2023	EPI_ISL_18007231
		A/large-billed_crow/Niigata/1502B004/2023	EPI_ISL_17949986
		A_common_kestrel_Korea_22WC597_2023_H5N1	EPI_ISL_18245382
		A_white-fronted_goose_Korea_22WC599_2023_H5N1	EPI_ISL_18245383
		A_white-fronted_goose_Korea_22WC252_2022_H5N1	EPI_ISL_18245332
		A/mute_swan/Chiba/1212001/2022	EPI_ISL_16955767
		A/chicken/Aichi/22A3T/2022	EPI_ISL_18284468
		A/Spot-billed_duck/Korea/K22–862–1/2022	EPI_ISL_15944665
		A/Spot-billed_duck/Korea/K22–856–2/2022	EPI_ISL_15944663
		A/Wild_bird/Korea/K22–742/2022	EPI_ISL_15943015
		A/Spot-billed_duck/Korea/K22–730–1/2022	EPI_ISL_15943002
		A_bean_goose_Korea_22WC079_2022_H5N1	EPI_ISL_18245303
		A/tundra_swan/Toyama/1611W001/2022	EPI_ISL_17949987
		A_white-fronted_goose_Korea_22WC116_2022_H5N1	EPI_ISL_18245307
		A_Em_Korea_22WF157–9P_2022_H5N1	EPI_ISL_18245277

Dataset	Trait	Strain	GISAID isolate ID
		A_white-fronted_goose_Korea_22WC324_2023_H5N1	EPI_ISL_18245344
		A/peregrine_falcon/Iwate/0301K004/2023	EPI_ISL_17309160
		A/large-billed_crow/Saitama/1101020/2023	EPI_ISL_18130247
		A/duck/Wakayama/22A1T/2022	EPI_ISL_18284467
		A/Spot-billed_duck/Korea/K22–920/2022	EPI_ISL_15944667
		A_white-fronted_goose_Korea_22WC244_2022_H5N1	EPI_ISL_18245328
H5 host (n = 35)	Wild duck	A/europian_wigeon/Kagoshima/KU-4/2023_(H5N1)	EPI_ISL_18529944
		A_gadwall_Korea_23WC215_2024_H5N1	EPI_ISL_20051141
		A/northern_pintail/Okayama/331A003/2023	EPI_ISL_18603584
		A_Eurasian_wigeon_Korea_23WS022–22_2023_H5N1	EPI_ISL_18717640
		A/Common_teal/Kagoshima/KU-6/2023_(H5N1)	EPI_ISL_18612263
		A_Northern_shoveler_Korea_23WC195_2023_H5N1	EPI_ISL_20051142
		A/mandarin_duck/Korea/WA875/2023(H5N6)	EPI_ISL_18819826
		A_Mandarin_duck_Korea_23WS033–1_2024_H5N6	EPI_ISL_20051143
	Goose	A/goose/Magadan/2272–5/2022	
		A/white-fronted_goose/Miyagi/0410D001/2022	EPI_ISL_15576616
		A/canada_goose/BC/AIVPHL-371/2023	
		A/white-fronted_goose/Korea/22WC328/2023	EPI_ISL_18245341
		A/white-fronted_goose/Korea/22WC365/2023	EPI_ISL_18245349
		A/white-fronted_goose/Korea/22WC254/2022	EPI_ISL_18245330
		A/white-fronted_goose/Korea/22WC252/2022	EPI_ISL_18245332
		A_Bean_goose_Korea_23WC111_2023_H5N6	EPI_ISL_18853650
		A_Bean_goose_Korea_23WC160_2024_H5N6	EPI_ISL_20051144
	Swan	A/whooper_swan/Hokkaido/0112Q105/2023	EPI_ISL_18740129
		A_Whooper_Swan_Korea_23WC069_2023_H5N1	EPI_ISL_20051146
		A_Whooper_Swan_Korea_23WC066_2023_H5N1	EPI_ISL_20051148
		A_Whooper_Swan_Korea_23WC068_2023_H5N1	EPI_ISL_20051147
		A_Whooper_swan_Korea_23WC117_2023_H5N1	EPI_ISL_20051145
		A/whooper_swan/Miyagi/0411B002/2023	EPI_ISL_18640911
		A_Whooper_swan_Korea_23WC116_2023_H5N6	EPI_ISL_18853651
		A_Whooper_Swan_Korea_23WC075_2023_H5N6	EPI_ISL_18853568
	etc	A/large-billed_crow/Hokkaido/0111Q100/2023	EPI_ISL_18640907
		A/goshawk/Gifu/1/2023	EPI_ISL_18740020
		A/white-naped_crane/Kagoshima/KU-13/2023_(H5N1)	EPI_ISL_18770562
		A/large-billed_crow/Osaka/2702A044/2024	EPI_ISL_19033312
		A/carrion_crow/Hokkaido/B079/2024	EPI_ISL_18876662
		A/large-billed_crow/Hokkaido/0111E092/2023	EPI_ISL_18640912
		A/large-billed_crow/Hokkaido/B093/2024	EPI_ISL_18968548
		A_Peregrine Falcon_Korea_23WC224_2024_H5N1	EPI_ISL_20051140
		A_great_cormorant_Korea_23WC229_2024_H5N6	EPI_ISL_20051139
		A/peregrine_falcon/Saga/4112A002/2023	EPI_ISL_18740267

Appendix Table 2. Analysis of mammalian adaptation markers in the eight gene segments of the isolated viruses.

Gene	Mutation	23 WS 022–22	23 WC 066	23 WC 068	23 WC 069	23 WC 075	23 WF 435	23 WC 111	23 WC 116	23 WC 117	23 WC 160	23 WS 033–1	23 WC 195	23 WC 215	23 WC 224	23 WC 229
PB2	271A	T	T	T	T	T	T	T	T	T	T	T	T	T	T	T
	292V	I	I	I	I	I	I	I	I	I	I	I	I	I	I	I
	526R	R	R	R	R	R	R	R	R	R	R	R	R	R	R	R
	588V	A	A	A	A	A	A	A	A	A	A	A	A	A	A	A
	591K	Q	Q	Q	Q	Q	Q	Q	Q	Q	Q	Q	Q	Q	Q	Q
	627K/V	E	E	E	E	E	E	E	E	E	E	E	E	E	E	E
	631L	M	M	M	M	M	M	M	M	M	M	M	M	M	M	M
	701N	D	D	D	D	D	D	D	D	D	D	D	D	D	D	D
PB1 (F2)	66S	**S**	**S**	**S**	**S**	**S**	**S**	**S**	**S**	**S**	**S**	**S**	**S**	**S**	**S**	**S**
PA	356R	K	K	K	K	K	K	K	K	K	K	K	K	K	K	K
HA	156A	**A**	**A**	**A**	**A**	**A**	**A**	**A**	**A**	**A**	V	V	**A**	**A**	**A**	**A**
	222L	N	N	N	N	N	N	N	N	N	N	N	N	N	N	N
NP	52N	H	H	H	H	Y	Y	Y	Y	H	Y	Y	H	H	H	Y
MP	95K	R	R	R	R	R	R	R	R	R	R	R	R	R	R	R

Appendix Table 3. Supported transmission routes of H5N1 between countries sharing the East-Asian wild bird migration flyway.

From	To	Bayes factor	Posterior probability	Actual migration rate
Northern Japan	South Korea	33.57	0.91	1.156
Outside of Korea	Northern Japan	25.453	0.89	1.146
South Korea	Central Japan	9.123	0.74	0.797
Outside of Korea	Central Japan	7.784	0.70	0.591
Northern Japan	Southern Japan	6.263	0.66	0.622
Northern Japan	Central Japan	6.104	0.65	0.951

Appendix Table 4. Supported transmission routes of H5N1 between geographic locations in South Korea and Japan

From	To	Bayes factor	Posterior probability	Actual migration rate
Japan	Gyeong-buk	41.24	0.926	1.096
Gyeong-buk	Jeon-buk	31.701	0.906	1.063
Jeon-nam	Jeju	8.406	0.720	1.159
Japan	Jeon-nam	8.245	0.716	0.73
Jeju	Jeon-nam	3.706	0.531	0.697

Appendix Table 5. Supported transmission of H5N1 between host types.

From	To	Bayes factor	Posterior probability	Actual migration rate
Japanese waterfowl	Japanese crow	46.186	0.934	1.726
Korean waterfowl	Korean domestic duck	13.376	0.803	0.966
Japanese waterfowl	Korean raptor	4.725	0.591	0.501
Japanese waterfowl	Korean waterfowl	4.116	0.557	0.611

Appendix Table 6. Supported transmission of H5N6 between geographic location

From	To	Bayes factor	Posterior probability	Actual migration rate
Jeon-nam	Gyeong-nam	24.176	0.850	1.51
Jeon-nam	Gyeong-buk	10.022	0.701	0.87
2022–2023 East Asia H5N1	Jeon-nam	8.635	0.669	0.43
Gyeong-buk	Jeon-buk	7.592	0.640	0.797
Jeon-nam	Japan	5.156	0.547	0.654
Gyeong-nam	Japan	4.392	0.507	0.593

Appendix Table 7. Supported transmission between host types.

From	To	Bayes factor	Posterior probability	Actual migration rate
2023–2024 Korean waterfowl	2023–2024 Japanese raptor	18.752	0.893	0.825
2023–2024 Korean waterfowl	2023–2024 Korean domestic duck	14.932	0.869	0.774
2022–2023 East Asia H5N1	2023–2024 Korean waterfowl	9.749	0.813	0.733

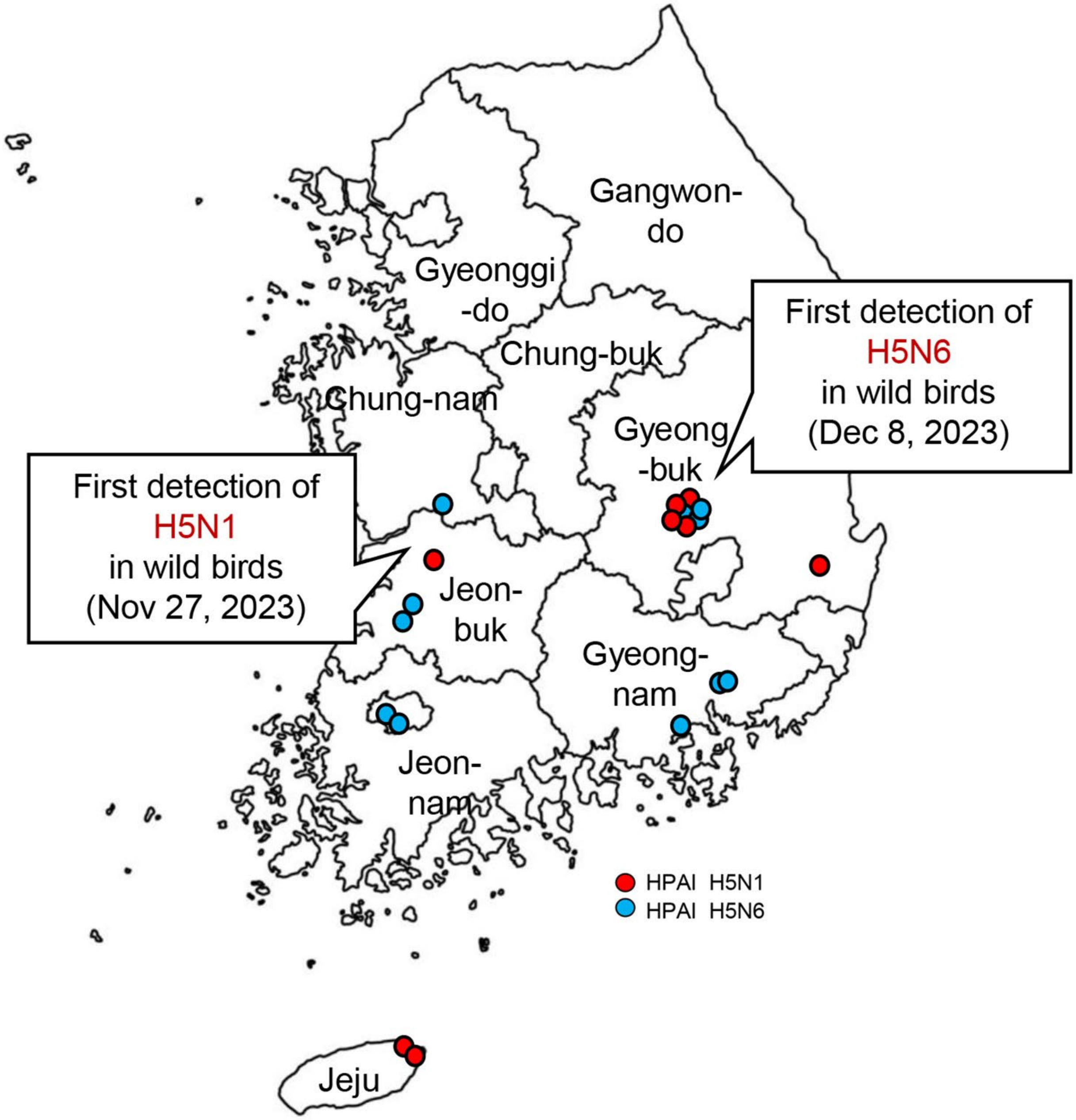

Appendix Figure 1. Geographic visualization of all detections of clade 2.3.4.4b highly pathogenic avian influenza A(H5N1) and (H5N6) viruses in wild birds in South Korea. First detection of each subtype is specified in text boxes. Red circles represent H5N1 detections and blue circles represent H5N6 detections in wild birds during November 2023 to February 2024.

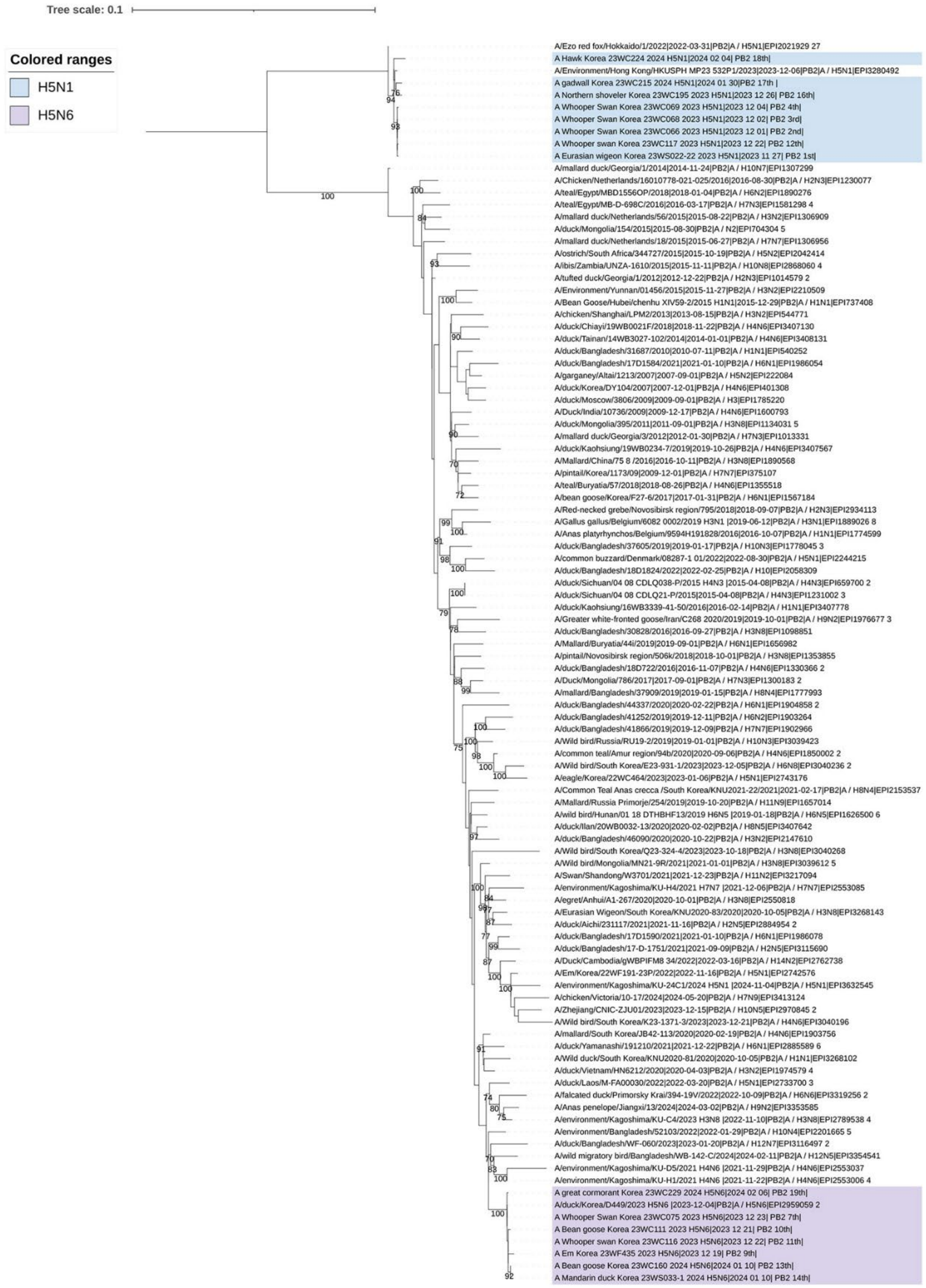

Appendix Figure 2. Maximum-likelihood tree constructed using PB2 from viral genomes of clade 2.3.4.4b H5N1 and H5N6 HPAIV isolated in this study. The numbers above the branches are bootstrap values. Monophyletic clades with a bootstrap support of 70% or higher were considered well-supported and showed. Scale bar indicates nucleotide substitutions per site.

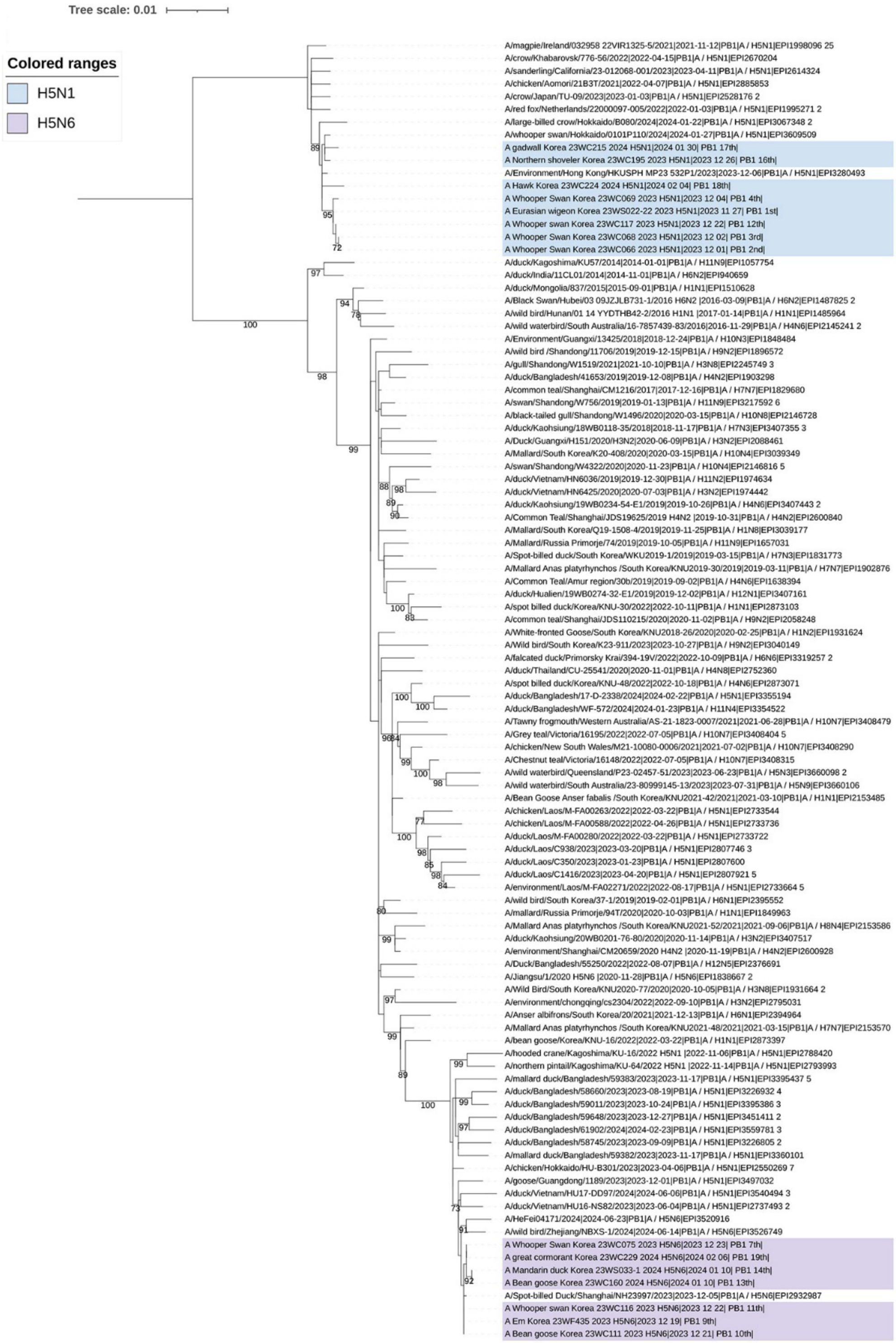

Appendix Figure 3. Maximum-likelihood tree constructed using PB1 from viral genomes of clade 2.3.4.4b H5N1 and H5N6 HPAIV isolated in this study. The numbers above the branches are bootstrap values. Monophyletic clades with a bootstrap support of 70% or higher were considered well-supported and showed. Scale bar indicates nucleotide substitutions per site.

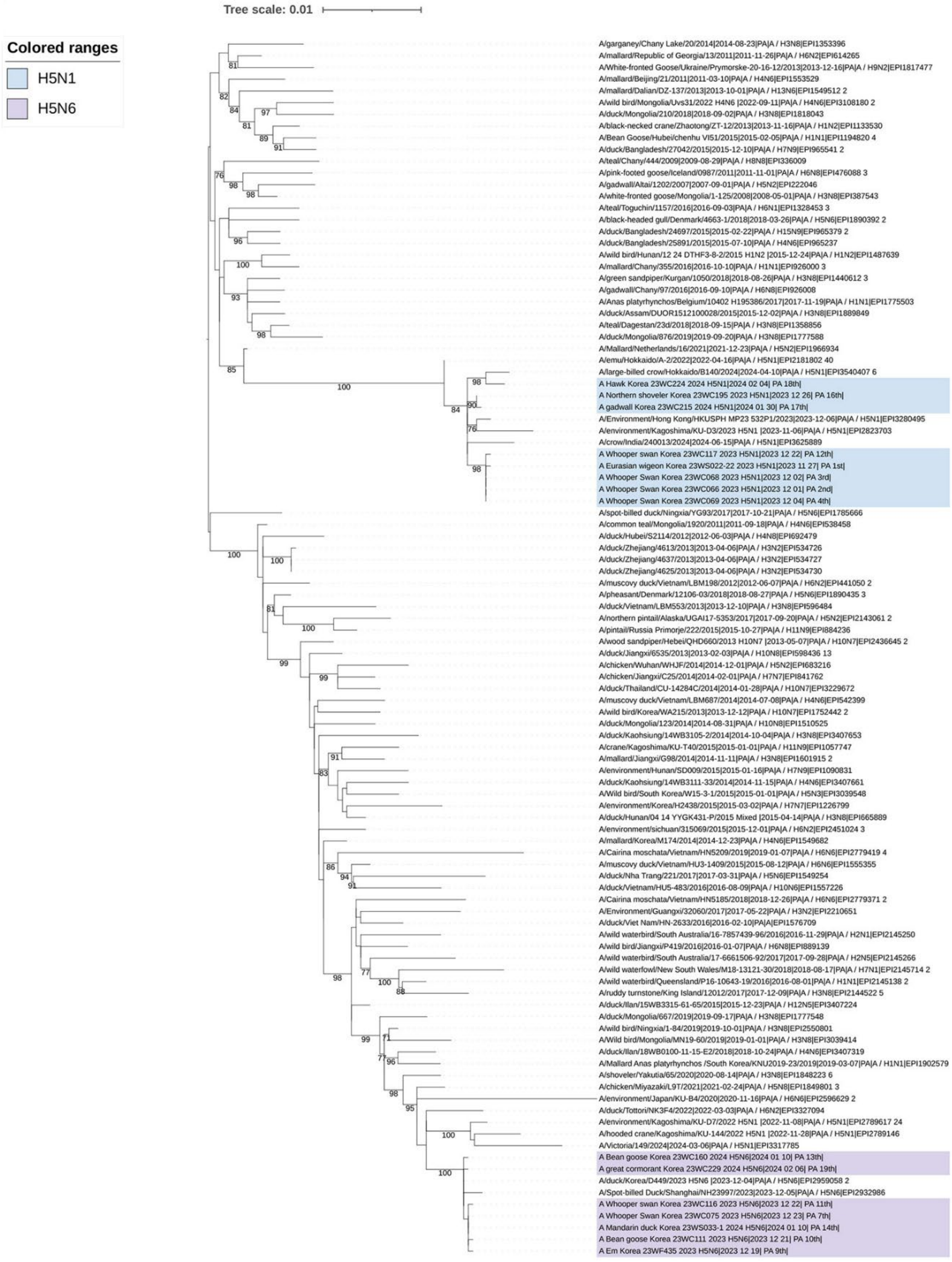

Appendix Figure 4. Maximum-likelihood tree constructed using PA from viral genomes of clade 2.3.4.4b H5N1 and H5N6 HPAIV isolated in this study. The numbers above the branches are bootstrap values. Monophyletic clades with a bootstrap support of 70% or higher were considered well-supported and showed. Scale bar indicates nucleotide substitutions per site.

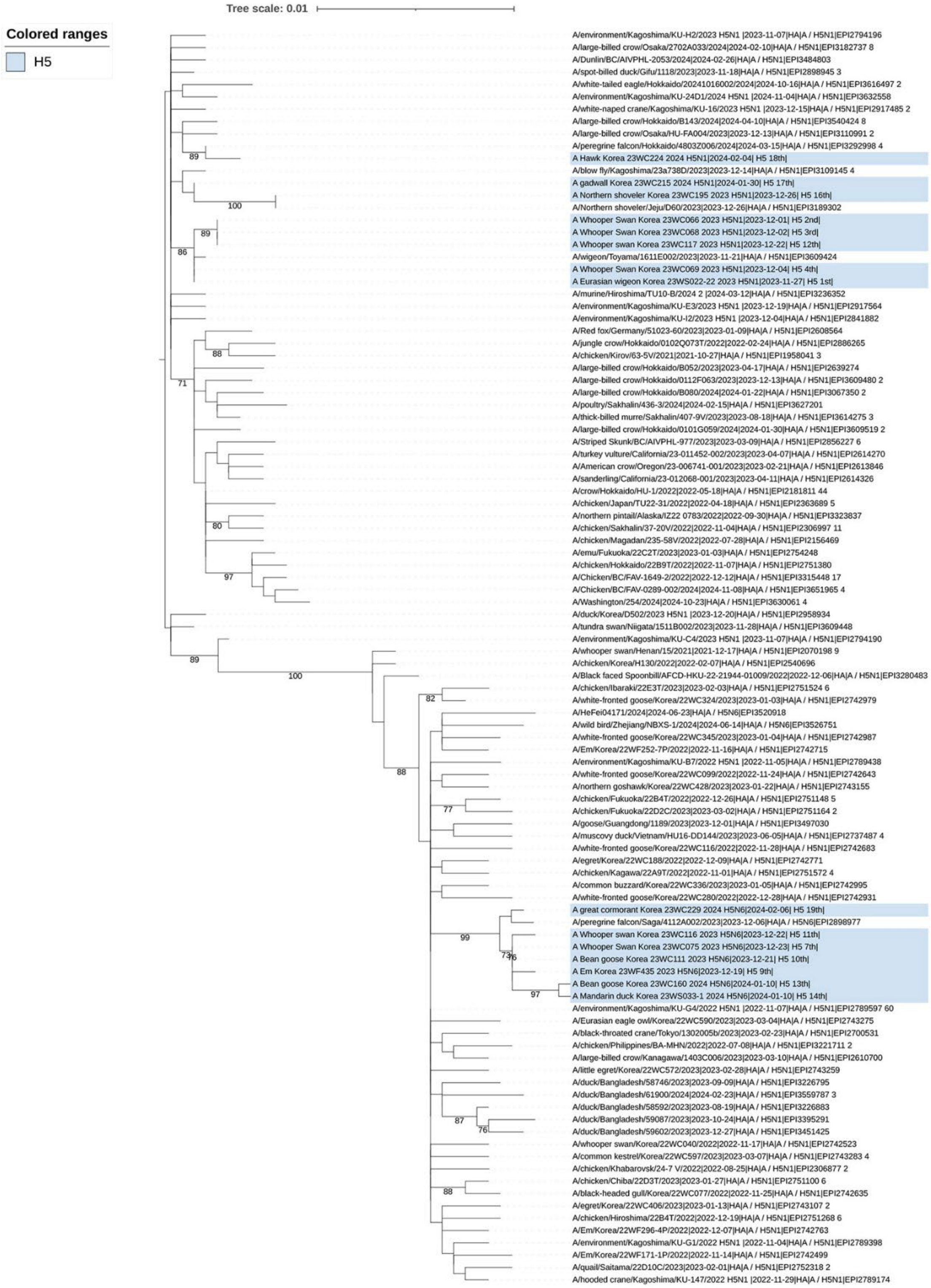

Appendix Figure 5. Maximum-likelihood tree constructed using HA from viral genomes of clade 2.3.4.4b H5N1 and H5N6 HPAIV isolated in this study. The numbers above the branches are bootstrap values. Monophyletic clades with a bootstrap support of 70% or higher were considered well-supported and showed. Scale bar indicates nucleotide substitutions per site.

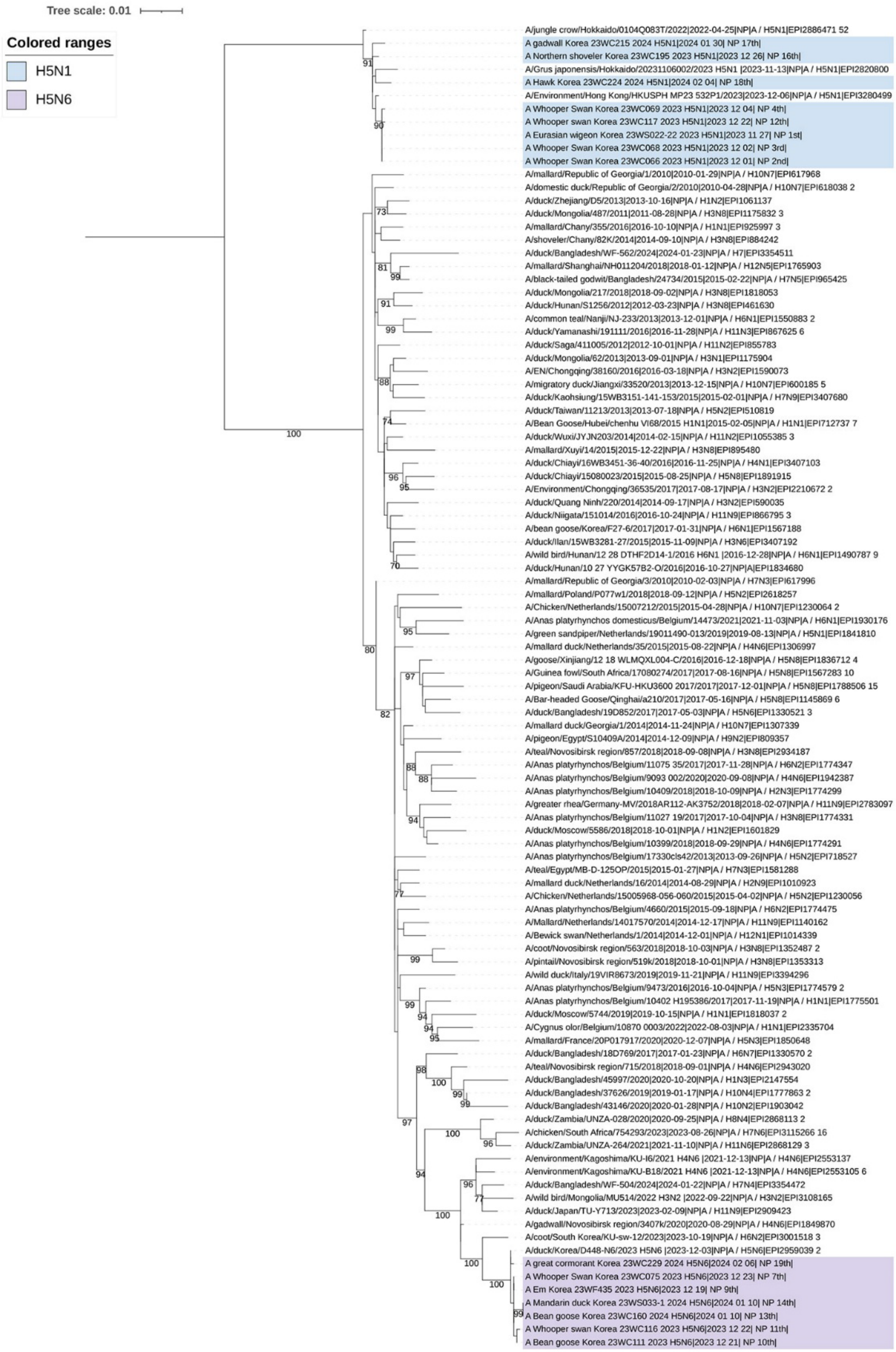

Appendix Figure 6. Maximum-likelihood tree constructed NP from viral genomes of clade 2.3.4.4b H5N1 and H5N6 HPAIV isolated in this study. The numbers above the branches are bootstrap values. Monophyletic clades with a bootstrap support of 70% or higher were considered well-supported and showed. Scale bar indicates nucleotide substitutions per site.

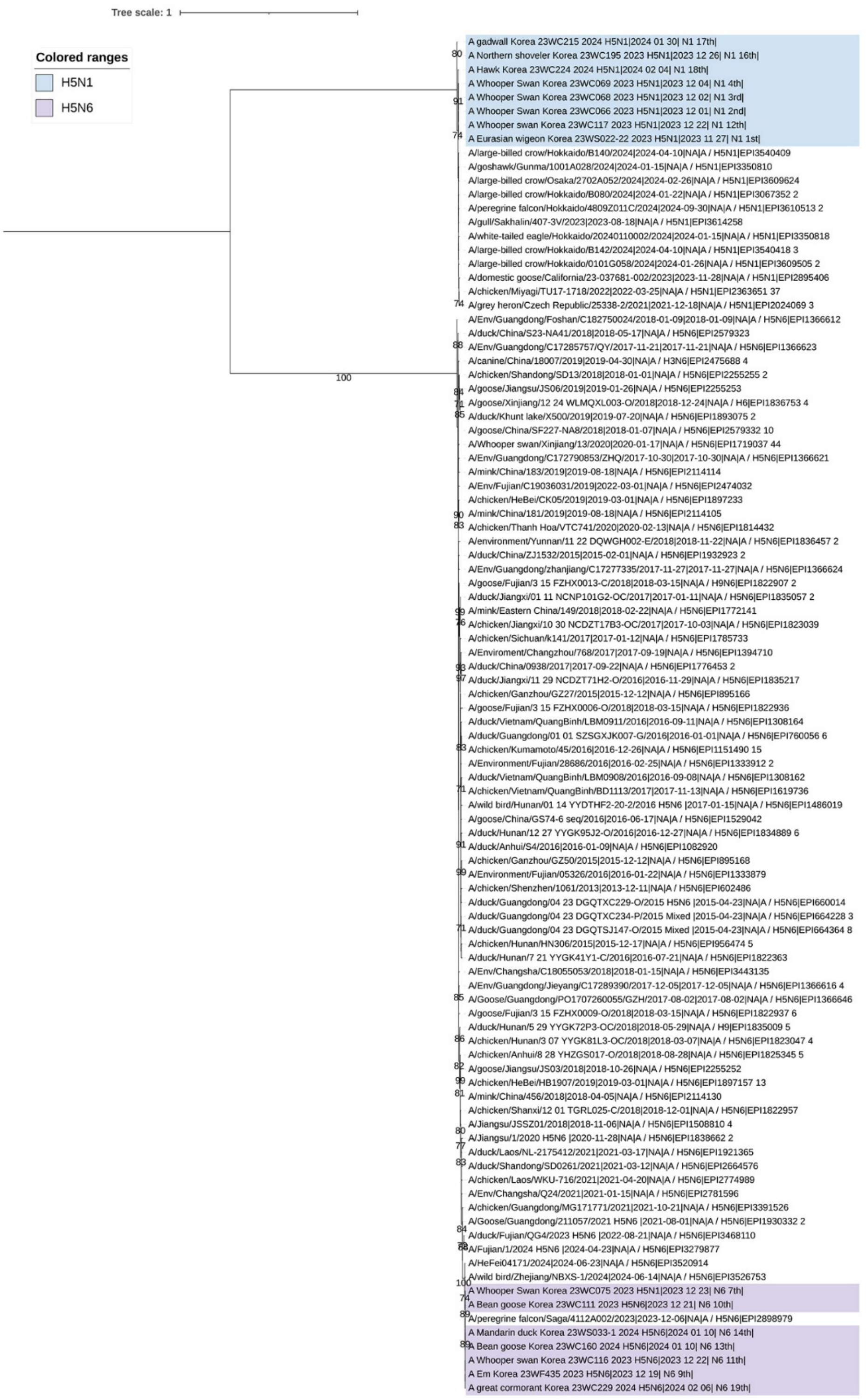

Appendix Figure 7. Maximum-likelihood tree constructed using NA from viral genomes of clade 2.3.4.4b H5N1 and H5N6 HPAIV isolated in this study. The numbers above the branches are bootstrap values. Monophyletic clades with a bootstrap support of 70% or higher were considered well-supported and showed. Scale bar indicates nucleotide substitutions per site.

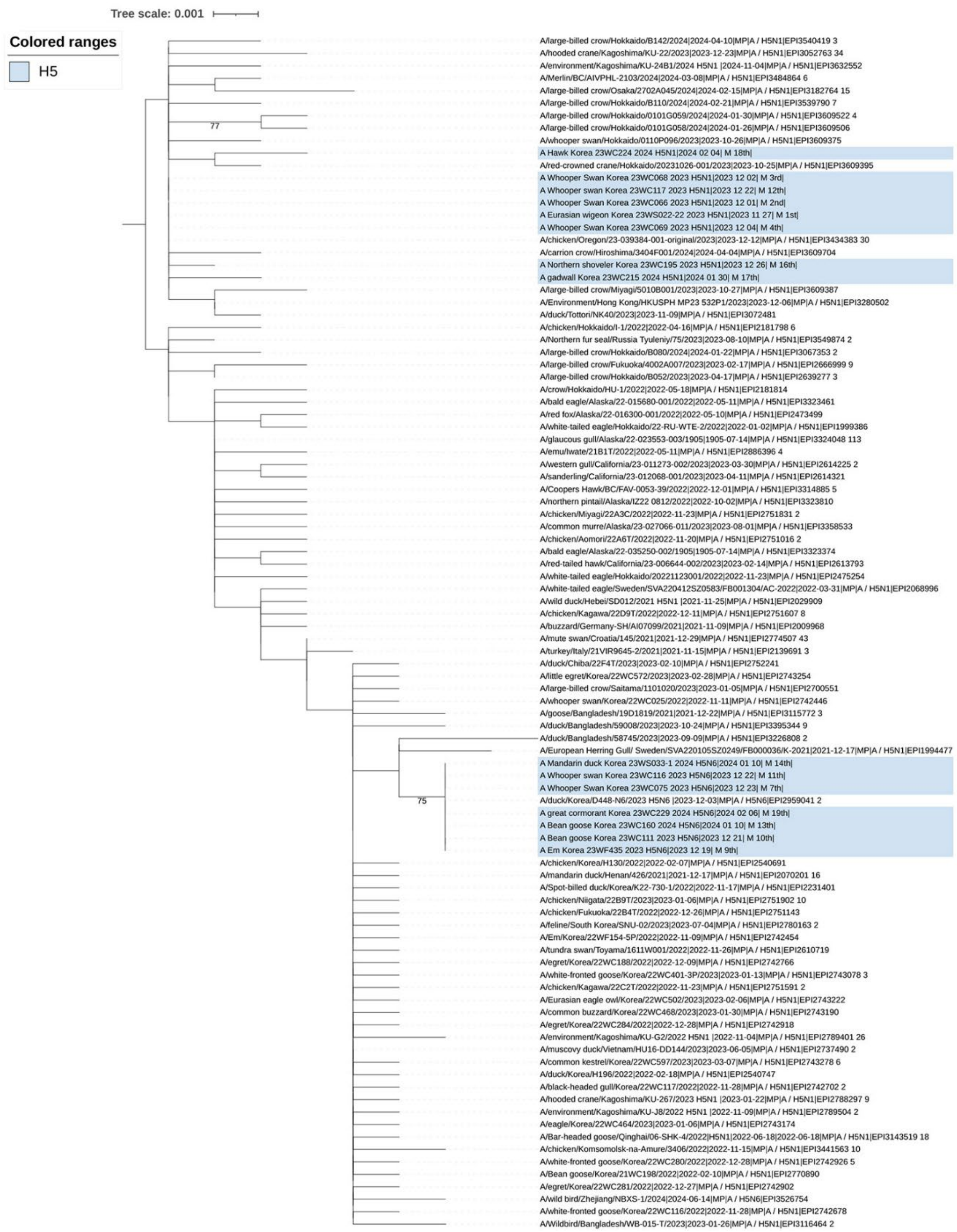

Appendix Figure 8. Maximum-likelihood tree constructed using MP from viral genomes of clade 2.3.4.4b H5N1 and H5N6 HPAIV isolated in this study. The numbers above the branches are bootstrap values. Monophyletic clades with a bootstrap support of 70% or higher were considered well-supported and showed. Scale bar indicates nucleotide substitutions per site.

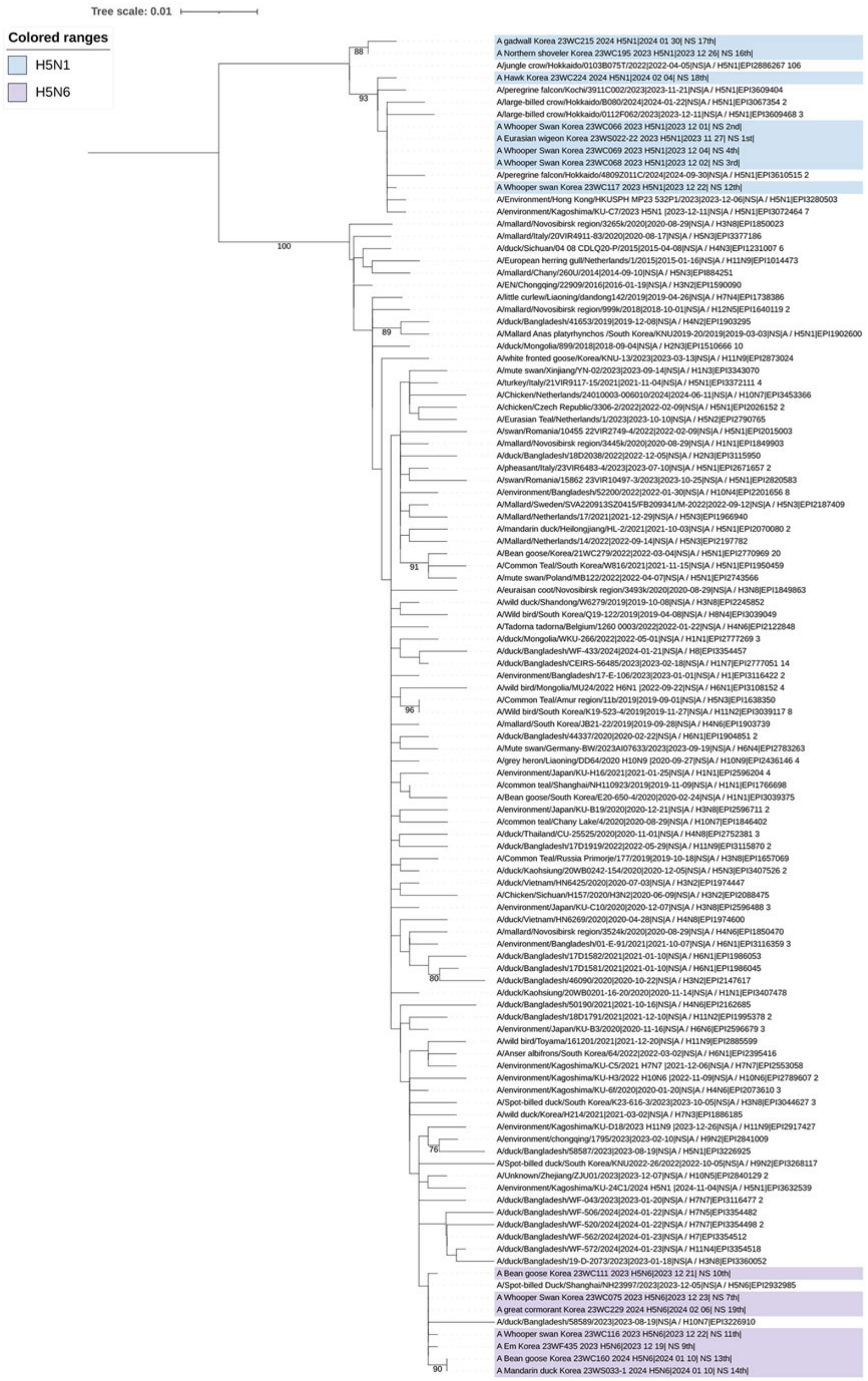

Appendix Figure 9. Maximum-likelihood tree constructed using NS from viral genomes of clade 2.3.4.4b H5N1 and H5N6 HPAIV isolated in this study. The numbers above the branches are bootstrap values. Monophyletic clades with a bootstrap support of 70% or higher were considered well-supported and showed. Scale bar indicates nucleotide substitutions per site.
